# The Interdependence between Schistosome Transmission and Protective Immunity

**DOI:** 10.3390/tropicalmed2030042

**Published:** 2017-08-23

**Authors:** Rebecca C. Oettle, Shona Wilson

**Affiliations:** Department of Pathology, University of Cambridge, Tennis Court Road, Cambridge CB2 1QP, UK; ro291@cam.ac.uk

**Keywords:** schistosomes, IgE, immunity, transmission dynamics

## Abstract

Mass drug administration (MDA) for control of schistosomiasis is likely to affect transmission dynamics through a combination of passive vaccination and reduction of local transmission intensity. This is indicated in phenomenological models of immunity and the impact of MDA, yet immunity parameters in these models are not validated by empirical data that reflects protective immunity to reinfection. There is significant empirical evidence supporting the role of IgE in acquired protective immunity. This is proposed to be a form of delayed concomitant immunity, driven at least in part by protective IgE responses to the tegument allergen-like (TAL) family of proteins. Specific questions have arisen from modeling studies regarding the strength and duration of the protective immune response. At present, field studies have not been specifically designed to address these questions. There is therefore a need for field studies that are explicitly designed to capture epidemiological effects of acquired immunity to elucidate these immunological interactions. In doing so, it is important to address the discourse between theoretical modelers and immuno-epidemiologists and develop mechanistic models that empirically define immunity parameters. This is of increasing significance in a climate of potential changing transmission dynamics following long-term implementation of MDA.

The age-infection intensity curve for all three major human-infecting schistosomes follows a pattern of increasing intensity through cumulative exposure until adolescence. After this infection intensities drop, irrespective of water exposure patterns [1]. It is therefore proposed that protective immunity is acquired with cumulative experience of infection. Once an individual has been exposed to a threshold of antigen, an effective immune response is mounted. Experience of infection is determined by either duration of infection or intensity of exposure. Since individuals in high-transmission areas are exposed to a higher level and greater diversity of antigens earlier in their lifetime, peak infection intensity is greater and occurs at an earlier age, when compared to areas of lower infection intensity [2]. This observation is termed the ‘peak shift’ [3,4]. Treatment with the antihelminthic praziquantel has been the cornerstone of morbidity control since the 1980s. Praziquantel kills adult schistosome worms, thereby providing passive immunisation through an antigenic stimulus delivered as adult worms die. Chemotherapy affects the development of individual protective immunity [4,5,6], but may also have the potential to considerably affect transmission at the community level [7].

Theoretical models are useful for policy making and can be used in both planning and evaluating the impact of control interventions on a broad epidemiological scale. For theoretical models of transmission to be useful they must have sufficient biological basis. The challenge of developing a schistosomiasis transmission model is in striking the balance between reality and parsimony. Schistosomiasis is highly focal, demonstrating significant spatial heterogeneity both between village and within village. This has been empirically demonstrated when studying the social, behavioural and cultural determinants of transmission on a microgeographical scale [8]. Likewise, immunological processes are studied on a microgeographical scale, relevant to the mechanisms and pathways that drive the development of immunity within populations studied. A model that aims to independently quantify the contribution of each modifying factor will be complex and prove challenging to draw useful analytical conclusions from [9,10]. Furthermore, model validity is likely to be restricted to the population against which the model is tested. Population and environmental heterogeneities may, however, play a significant role in dictating transmission dynamics [11].

The computational predictions of mathematical models are only as good as the data against which they are calibrated and validated. Models are based upon the existing knowledge of biological processes and the data available to describe and quantify these interactions [12]. Recent studies continue to base the dynamics of infection solely on the explanation of variable exposure with age [13,14,15], despite considerable field evidence to support the role of acquired immunity. It is important that models evolve alongside the emergence of experimental evidence supporting model parameterization [16]. When immunological concepts have been modeled, immune processes have typically been described phenomenologically, with interactions based on assumptions with limited empirical backing [12]. A shortage of field data to inform quantitative estimates for parameter values and limits to theoretical understanding have frequently been cited as reasons for not exploring the contribution of immunity to transmission dynamics in more detail [13,17]. Furthermore, immuno-epidemiological studies have not historically been designed to provide empirical evidence for parameter values directly [16,17].

## 1. Required Immunological Parameters

“A closer collaboration between biometricians and parasitologists, and a better acquaintanceship of each with the methods of the other, is one of the most useful things we can work for today” [18,19].

When studying the dynamics of infectious diseases interdisciplinary collaboration is essential. This is particularly important in cases of such complex social and ecological interactions as schistosomiasis. It is therefore important to identify specific areas where there is a greater need for quantitative estimates of model parameters. This is especially important with the recent clear shift from a focus on control towards discussion of elimination efforts. Subsequently, a number of recent models have attempted to predict the requirements and impacts of mass drug administration (MDA) in reaching proposed elimination targets [13,20,21].

It has been argued that models that do not incorporate the dynamics of acquired immunity lack power in reproducing age-intensity profiles [13,22] and may overestimate the positive impact of control achieved by MDA [23,24]. This would occur if reduced transmission delays the development of immunity. Existing transmission models incorporating protective immunity suggest that worm lifespan and the rate of immune decay significantly contribute to the magnitude of specific antibody response [5,20]. Moreover, as many communities have now experienced access to mass drug administration with praziquantel, to a varying degree [25], fluctuations in antibody magnitude are likely to be occurring. There is therefore a need for field studies that are explicitly designed to capture epidemiological effects of acquired immunity. This will aid model development through provision of parameter estimates for transmission models [9].

The following gaps in our theoretical understanding and data requirements have been highlighted in phenomenological examination of immune responses, some of which relate to immuno-epidemiological study design, some specifically to immune mechanisms:

- Information on the demographic profile of communities from which immunological cohorts are drawn [15].
- Longitudinal studies of age-specific correlations between parasite burden and immune responses, incorporating individual data from a full range of age classes, in addition to comparison of communities with differing overall levels of endemic infection [13,26].
- Better knowledge of patterns of exposure experienced by immuno-epidemiological cohorts [26], requirement for water contact studies including cultural and sociological analysis [8].
- Specific analysis of age-dependent immunological parameters, including immunological responsiveness (strength of protective response) and loss of immunological memory [5,20,26], with particular reference to the parameterisation of protective immunity.
- Effects of inappropriate responses such as blocking antibodies and parasite-induced immunomodulation [26].
- The impact of immune responses that affect parasite fecundity and host excretion of eggs [20,26].

## 2. Delayed Concomitant Immunity

Mechanisms that confer partial immunity to schistosomes are not fully understood due to reliance on immuno-epidemiological evidence, with the murine experimental model being largely inappropriate for modelling human resistance to infection. However, levels of IgE specific to schistosome antigens, particularly antigens derived from the adult worm (SWA), are negatively associated with reinfection in studies examining all three major human-infecting schistosomes [27,28,29].

While historically a switch in the balance between SWA-IgE and the blocking antibody SWA-IgG4 was proposed as the key to immunity, with adults having a greater SWA-IgE:IgG4 ratio than children [30,31], the current leading hypothesis of the mechanism of immunity is that exposure to worm death is required for the development of immunity [2,6]. This has been referred to as delayed concomitant immunity [32], a development on the concomitant immunity first described by Smithers and Terry, in which established live worms mediate an immune response that prevents establishment of further parasites [33]. In the model of delayed concomitant immunity, the source of antigen to which the IgE is raised is the adult worms, upon worm death, while the focus of immune attack remains the early schistosomule. From an evolutionary point of view, this represents a stand-off between the parasite and the host—sufficient time is awarded to the parasite to produce the next generation, while the host is protected against the build-up of an excessive worm burden. Biologically this hypothesis is also compelling, with the adult worm being a master of immune evasion, successfully living within the blood stream for an estimated 3.5–10.5 years depending on species [34,35], while the transformation undergone during the transition from free-living cercaria to host-dwelling schistosomule provides a point of vulnerability within the parasite’s life history for the host to attack.

**Figure 1:**
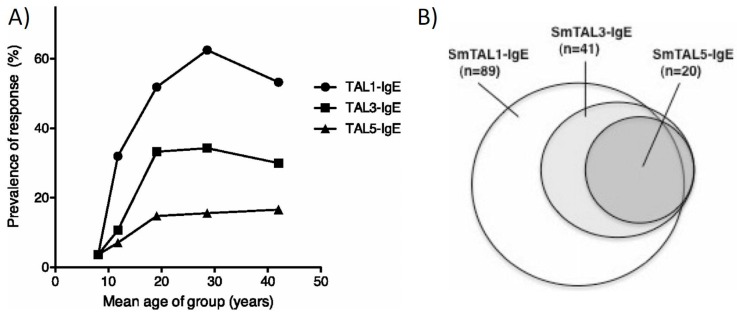
IgE Responses to SmTAL1, SmTAL3 and SmTAL5. Shown are the 9-week post-treatment IgE responses of a cohort of males, aged 7–60 years, resident in a Ugandan fishing village of high endemicity. (**A**) Prevalence of detectable IgE specific to SmTAL1, SmTAL3 and SmTAL5 by age; (**B**) Venn diagram displaying sequentiality of detectable of IgE specific to SmTAL1, SmTAL3 and SmTAL5. Figures are reproduced with permission from [32].

This hypothesis of delayed concomitant immunity is best described through the responses to members of the *Schistosoma mansoni*–tegumental allergen-like (SmTALs) protein family. SmTAL1 (previously Sm22.6), the first of the family to be described, is the major IgE antigen detected on western blots probed with sera from endemic populations [29]. SmTAL1-IgE levels rise with age (Figure 1a), commensurate with the drop in infection intensities in adolescence, and have been shown to be negatively associated with reinfection intensities [36,37]. SmTAL1 is expressed from the 24 h schistosomule onwards, mainly within the tegument of the adult worm, where it is not substantially exposed to the host by live worms. IgE levels to SmTAL1 increase in the weeks after praziquantel treatment, confirming increased exposure of the antigen to the host during worm death [38]. SmTAL3, also expressed within the adult worm, follows a similar increase in detectable IgE with age, but with SmTAL3-IgE responders being a subset of SmTAL1-IgE responders (Figure 1b) [32,37]; thus, it is proposed that a greater exposure to dying worms is required for a SmTAL3-IgE response. If true, this would be illustrative of an antigen-threshold effect occurring for IgE responses to antigens of this family.

Responsiveness to SmTAL2, which is expressed throughout the life cycle, offers an illustrative counterpoint to SmTAL1/3 responses. An IgE response to this protein is measurable, but only amongst the very youngest members of populations in endemic areas, ultimately being regulated by IgG4 [37,39]. The constant exposure to SmTAL2 from dying eggs within the tissue, in contrast to the intermittent exposure to SmTAL1 and SmTAL3 from the adults, makes it imperative that the host down-regulates the potentially damaging IgE response to SmTAL2, a down-regulation that is not required for the members of the family cryptically expressed in adults. However, with SmTAL1 and 3 not expressed in the earliest schistosomule stages, as would be required if this stage is the target of immunity, IgE response to SmTAL1 and SmTAL3 will not be explanatory of immunity, and indeed after controlling for age, IgE responses to these antigens are not significantly associated with immunity [32].

Immunity via exposure to dying adult worms is not easily reproduced in murine models, with long-term egg deposition altering vasculature, and hence migratory pathways of worms infecting from trickle exposures. Immuno-epidemiological studies suggest a slow-developing IgE-mediated mechanism, rather than sentinel myeloid cell-derived regulatory immune responses, as seen in a multiple (pre-egg laying) cercarial challenge murine model [40]. It is therefore hypothesised that skin resident mast cells would be the first responders in immune individuals [41]. This will dramatically alter the immune profile of the response upon cercarial challenge from one of regulation to one characterised by IgE mediated by type-1 hypersensitivity reactions. Potential targets include SmTAL5, for which age-dependent IgE responsiveness is observed (Figure 1a) [37]. Expressed in the early schistosomules, SmTAL5 displays IgE cross-reactivity with SmTAL3, with SmTAL3 appearing to be the antigenic source for this IgE response [32]. It is this cross-reactivity between the adult worm expressed SmTAL3 and the early schistosomule expressed SmTAL5 that is proposed to exert the delayed concomitant immunity, though it is unlikely that this is the sole cross-reactive antigen that is involved in the development of partial immunity.

Eosinophil infiltration has also been implicated in protective human IgE responsiveness. In vitro eosinophils can mediate killing of schistosomules in an IgE-mediated manner [42]. In *S. haematobium* infection, eosinophils from Gambian individuals with the greatest expansion in eosinophil number have the greatest capability to kill schistosomules [43], and in adult Kenyan car-washers infected with *S. mansoni*, both eosinophil numbers, and their expression of the tetrameric high-affinity IgE receptor on their surface, are associated with immunity to reinfection [44]. It is tempting to hypothesise that cross-linking of IgE on mast cells leads to chemotactic infiltration of eosinophils, which then release their granular contents to kill the schistosomules, but without a valid model of IgE-armed skin responses this remains a hypothesis.

## 3. IgE Responses and Praziquantel Treatment

The proposed reliance on worm death for development of protective immunity to schistosomes suggests that treatment with praziquantel could provide a ‘passive vaccination’ effect, boosting the development of immunity. This passive vaccination effect is eloquently illustrated in the studies of car-washers conducted in Kisumu, Kenya, in which praziquantel treatment was administered after every detectable *S. mansoni* reinfection. As the number of treatments administered increased, the longer the observed duration to reinfection [45]. In comparative studies enrolling longer-term exposed sand-harvesters, this immunity was observable after two treatments, but an extended process of 7 infections and treatment was required for car-washers who were more recently exposed to infection. In agreement with our hypothesis, at baseline the greater exposed sand-harvesters had greater circulating levels of SmTAL1-IgE, indicating that this group had moved further along the path towards immunity prior to the commencement of the study [46]. During the reinfection-treatment cycles, elements of the immune system involved in IgE production, in particular soluble and B cell membrane-bound levels of CD23, the low-affinity IgE receptor, became elevated in comparison to baseline [47]. The elevation of these immune elements was also observed in a cohort of schoolchildren treated four times annually [48].

Immunologically, elevated levels of SWA and SmTAL1-, 3- and 5-specific IgE are observed 3–9 weeks post-treatment. These post-treatment IgE levels are better correlates of resistance to reinfection as measured 1–2 years post-treatment, than pre-treatment antibody levels [32,38,49]. This boost in IgE to adult worm-derived antigens, and antigens cross-reactive with worm-derived antigens, is accompanied by an increase in histamine release upon antigen stimulation of basophils, indicating that the increased IgE is biologically active [50]. The elevated specific IgE levels are also accompanied by an increase in immune cell proliferation [51] and production of type-2 immune cytokines, such as IL-4, 5 and 13 by peripheral blood mononuclear cell and whole blood cultures [52,53]. IL-5, an eosinophil maturation factor, in particular, is elevated in post-treatment supernatants of SWA stimulated immune cells [54,55]. For Ugandan children residing in high-transmission fishing villages, the pre-treatment strength of this IL-5 cytokine response is associated with levels of SWA-IgE measured post-treatment with praziquantel [56]. Mirroring this ex vivo IL-5 response are circulating 24 h post-treatment IL-5 levels measured in the plasma [57]. For *S. haematobium* infection, the IL-5 levels detected 24 h post-treatment are associated with pre-treatment SWA-IgE and eosinophil levels. Furthermore, post-treatment IL-5 is associated with pre-treatment infection intensity, indicating that both the primed state of the immune system, or prior exposure to dying worms, and the amount of antigen the system is exposed to upon treatment, is crucial in generating this IL-5 response. In turn, 24 h post-treatment levels of IL-5 are positively associated with protective post-treatment SWA-IgE levels and elevated circulating eosinophil numbers [58].

## 4. Rate of Decay of Protective Immunity

What is clear from the above immunological evidence is that treatment with praziquantel can give a boost to proposed elements of a protective immune response; that is, type 2 cytokine responsiveness, levels of specific IgE and eosinophil numbers. Moreover, this could be providing a ‘passive vaccination’ effect, allowing individuals in endemic areas to develop their immunity at an earlier age. While this is encouraging, as stated earlier, when conducting reinfection studies immuno-epidemiologists work on a microgeographical scale and tend to seek out study sites with high transmission, or particularly highly-exposed occupational cohorts, as this allows us to examine correlates of immunity. Little is known about whether a significant vaccination effect occurs if transmission is low or, as is the aim with multifaceted control strategies, driven to be being very low. Successful control programs have, over time, altered the distribution of schistosomiasis. French et al. [59,60], noted a change in force of infection following repeated MDA campaigns in five countries where the Schistosomiasis Control Initiative has been working for the past decade. In an era where control is primarily based on upscaling chemotherapy, it is increasingly important to understand the impact of MDA on transmission dynamics. Are the levels of antigen exposure induced by annual treatment going to be sufficient to drive a vaccinating effect? Or indeed, by driving transmission to a point when it is very low, but continuing, will antigen exposure be limited to the point that it takes into late adolescence or early adulthood for antigen thresholds to be passed and immunity to have fully developed? Early modeling studies indicate that mass chemotherapy may reduce immunity at the community level, to the extent that, following cessation of control, parasite load in the older age classes may rise above the pre-control burden [7]. The latter will have implications for school-based strategies if the focus of control moves further from public health morbidity control strategies towards interruption of transmission.

Mitchell et al. [20] related protective immunity to reduced egg production, a phenomenon observed in *S. haematobium* field studies, but not seen for *S. mansoni* infections [61,62]. It was concluded that MDA will initially boost protective immunity, but antibody levels could decline below pre-treatment levels during or after cessation of MDA. The model is calibrated on short-term pre- and post-treatment studies of *S. haematobium* infection, but does support the hypothesis that MDA may disrupt the build-up of protective immunity. They also proposed that if reduced transmission led to reduced antigen exposure, antibody levels will be strongly influenced by immune decay rates and demonstrated a rebound in egg excretion following cessation of MDA activities [20]. It is important to note that this paper does not examine protective response against reinfection. Anti-fecundity and anti-infection immunity are likely mediated by different antibody classes [62], therefore have differing mechanisms of induction and preservation by the memory component of the immune system.

In relation to the immune decay rate of IgE, biologically-active IgE may be sustained for months after exposure to the initial stimuli of production due to long-lived IgE plasma cells maintaining IgE production [63]. Yet, when we conducted a longitudinal study looking at immune factors when annual praziquantel treatment of Kenyan school-aged children was combined with biannual mollusciding of the one source of transmission in the area, the levels of circulating SWA-IgE were significantly lower one and two years after the baseline survey (unpublished results). This suggests that antigen exposure is required to maintain production of SWA-IgE at pre-treatment levels. Basophil bioassays were not conducted so we do not know whether SWA-IgE was reduced to a level sufficiently low to prevent cross-linking of IgE bound to receptors on effector cells. At the same time-points, however, whole blood cultures produced elevated SWA-specific type-2 cytokines, indicating that T cell memory is robust and maintained over that extended period [64]. Although it is thought that B cells switched to IgE are very poorly recruited to the memory compartment of the immune system [63,65], it has recently been shown in murine models of peanut allergy that a memory response is crucial to re-activation of clinical IgE reactivity, with IL-4 from T cells driving IgG memory B cells to switch and mature into IgE plasmablasts [66]. In humans, grass pollen allergic rhinitis sufferers have higher numbers of circulating memory B cells that proliferate upon allergen exposure than control subjects [67]. It is therefore plausible that if transmission is still occurring, the memory component of the immune response could maintain or re-establish protective IgE-mediated immunity upon intermittent exposure to worm death, the long-term dynamics of which will depend on the local transmission patterns.

## 5. Variation in Estimates of Immune Parameters

An important consideration is that numerical analyses are necessarily based upon a restricted set of parameter combinations [26]. These broad parameter estimates are likely to vary between locations [68], with authors highlighting the importance of using locality-specific values to generate predictions from models [69]. The generalisability of these models from one situation to another is consequently restricted [12]. In particular reference to immunological parameters, Chan et al. [68] argue that immuno-epidemiological studies on the individual community level fail to capture the true dynamics of transmission as a result of variability in parasitological examination and antibody titres. They therefore proposed the use of mathematical models to inform the design of field studies, a concept further discussed in relation to ecological modelling by Restif et al. [16]. Chan et al. [68] advocate that model-guided design of fieldwork can strengthen the power of data analysis and inferences. They also suggest that the village is the smallest unit considered when studying patterns of transmission. Immunologically, this leads to the technical hurdle of scale; however, advancing technology for sample processing, including liquid handling capabilities and associated reductions in sample volume requirements, are now such that the potential to conduct serologically-based immunological studies on a wider scale does exist.

We believe, given the field-based evidence, that more mechanistic modelling of immunity in future schistosome transmission models is required. We have provided discussion, from an immunological perspective, on what is known from existing field data, but acknowledge that previous immunology studies, through scale and design, may fail to capture the parameters that modelers identify as important. However, with technological advances, an integrated approach between theoretical modelers and field immunologists is now possible and of utmost importance if transmission models are to truly capture schistosome infection dynamics within endemic areas.
